# Multiclass Segmentation of Breast Tissue and Suspicious Findings: A Simulation-Based Study for the Development of Self-Steering Tomosynthesis

**DOI:** 10.3390/tomography9030092

**Published:** 2023-06-10

**Authors:** Bruno Barufaldi, Yann N. G. da Nobrega, Giulia Carvalhal, Joao P. V. Teixeira, Telmo M. Silva Filho, Thais G. do Rego, Yuri Malheiros, Raymond J. Acciavatti, Andrew D. A. Maidment

**Affiliations:** 1 Department of Radiology, University of Pennsylvania, Philadelphia, PA 19104, USA; racci@pennmedicine.upenn.edu (R.J.A.); andrew.maidment@pennmedicine.upenn.edu (A.D.A.M.); 2 Center of Informatics, Federal University of Paraiba, Joao Pessoa 58051-900, PB, Brazil; yann.nicholas@gmail.com (Y.N.G.d.N.); carvalhalgiulia@gmail.com (G.C.); joaoteixeira@eng.ci.ufpb.br (J.P.V.T.); thais@ci.ufpb.br (T.G.d.R.); yuri@ci.ufpb.br (Y.M.); 3 Department of Engineering Mathematics, University of Bristol, Bristol BS8 1QU, UK; telmo.silvafilho@bristol.ac.uk

**Keywords:** digital breast tomosynthesis, virtual clinical trials, artificial intelligence, risk stratification

## Abstract

In breast tomosynthesis, multiple low-dose projections are acquired in a single scanning direction over a limited angular range to produce cross-sectional planes through the breast for three-dimensional imaging interpretation. We built a next-generation tomosynthesis system capable of multidirectional source motion with the intent to customize scanning motions around “suspicious findings”. Customized acquisitions can improve the image quality in areas that require increased scrutiny, such as breast cancers, architectural distortions, and dense clusters. In this paper, virtual clinical trial techniques were used to analyze whether a finding or area at high risk of masking cancers can be detected in a single low-dose projection and thus be used for motion planning. This represents a step towards customizing the subsequent low-dose projection acquisitions autonomously, guided by the first low-dose projection; we call this technique “self-steering tomosynthesis.” A U-Net was used to classify the low-dose projections into “risk classes” in simulated breasts with soft-tissue lesions; class probabilities were modified using post hoc Dirichlet calibration (DC). DC improved the multiclass segmentation (Dice = 0.43 vs. 0.28 before DC) and significantly reduced false positives (FPs) from the class of the highest risk of masking (sensitivity = 81.3% at 2 FPs per image vs. 76.0%). This simulation-based study demonstrated the feasibility of identifying suspicious areas using a single low-dose projection for self-steering tomosynthesis.

## 1. Introduction

Digital breast tomosynthesis (DBT) uses a limited angle of acquisition with a small number of low-dose projections acquired in a single left–right scanning motion to produce cross-sectional planes through the breast for three-dimensional data visualization. Today, DBT is considered the state-of-the-art technique for screening, demonstrating increased sensitivity in cancer detection and lower recall rates as compared to digital mammography (DM) [1,2]. Despite the substantial technological advancements in DBT, clinical tomosynthesis systems are not autonomous; the scanning orbit is not customized based on suspicious findings or other imaging biomarkers. Instead, the scanning orbit follows the same left–right motion [3] in every patient, regardless of breast size or internal composition.

The University of Pennsylvania has developed a prototype next-generation tomosynthesis (NGT) system with more complex scanning motions than current clinical DBT systems [4,5,6,7]. The NGT system is capable of scanning with an additional component of source motion in the posteroanterior direction (perpendicular to the conventional motion), reducing out-of-focus structures [7]. Our ultimate goal is to incorporate scanning motions into the NGT system that are customized around suspicious areas (e.g., breast cancers, parenchyma distortions, and dense clusters), as well as areas prone to cancer masking, for improved breast screening and diagnosis. For that, the NGT requires the acquisition of a single low-dose projection (i.e., a scout image) that is processed to identify the suspicious areas precisely, and to determine in real time the subsequent projections acquired autonomously during the scanning motion.

Although the NGT system is not yet capable of customizing its scanning acquisitions autonomously, we wanted to demonstrate the feasibility of identifying suspicious areas using low-dose projections. This work represents an important step in that direction by proving that findings or areas prone to masking can be identified in a single projection; the information acquired from the low-dose image acquired at the start of the scan could ultimately be used to guide the remainder of the scanning motion (“self-steering tomosynthesis”). Since the NGT is not being used clinically yet, the feasibility of a self-steering scanning motion was investigated in this work using virtual clinical trial (VCT) methods.

VCT methods have been used for the optimization of the NGT system [8,9] as a cost-effective alternative to conducting clinical trials, allowing researchers to answer fundamental questions using in silico simulations; VCTs can prototype and replicate clinical trials [9,10] by making available a plethora of evidence-based data for regulatory approval of novel imaging systems [10]. VCTs are targeted toward specific “tasks”, usually requiring the complex simulation of human anatomy (anthropomorphic phantoms) in order to answer clinical questions [8].

In our previous work, a Perlin-based phantom [11] was developed to simulate X-ray images of complex breast parenchyma in DBT. In a follow-up study by da Nobrega et al., Perlin-based phantoms were used to train a U-Net model to segment various classes of tissue (for example, adipose and dense tissue, as well as lesions), offering preliminary data suggesting that a low-dose projection could indeed guide the remainder of the scan [12]. However, the U-Net model resulted in a high rate of false positives, especially for regions of suspicious findings. A high number of false positives reduces the specificity of the detection method, potentially complicating the development of customized scanning motions for self-steering tomosynthesis.

Probability calibration can be used to reduce the false positive rates of multiclass classifiers. In accordance with the main concept of calibration [13], a multiclass probabilistic classifier should only be considered well-calibrated if instances of a particular class receive probabilities in accordance with the actual class distribution of the data. For example, if we have amongst the test instances a predicted probability vector *s* = [0.1, 0.2, 0.7], the class distribution of *s* should be approximately 10%, 20%, and 70% for the first, second, and third classes, respectively. This is a fundamental property when using a classifier for cost-sensitive classification or for human decision making, because a calibrated classifier correctly quantifies the level of uncertainty or confidence associated with its predictions [13].

Bringing this concept to our scenario, a U-Net segmentation corresponds to a classifier prediction at the pixel level of an image. We can directly apply well-known techniques to visualize (e.g., reliability diagrams) [14] and measure (estimated calibration error or ECE) [15] the current state of calibration of our segmentation model. In this study, we used a calibration method (Dirichlet calibration or DC) to adjust the class probabilities predicted by a U-Net model trained with VCT-based data for the assessment of risk. The U-Net model is proposed as a method for detecting suspicious findings or areas prone to masking in a single projection image. This work ultimately has applications in developing task-directed scanning motions for DBT.

## 2. Materials and Methods

### 2.1. Perlin-Based Phantom and Lesion Simulation

A principal component analysis (PCA)-based method was used to simulate the outlines of breasts under mammographic compression [16,17,18]. The outlines were created using a compressed breast thickness (CBT) of 30–70 mm and a chest wall to nipple distance (CND) of 50–110 mm. Coarse tissue (adipose and dense) was embedded into the breast outlines using a recursive partitioning algorithm [19]. Finally, 3D fractal noise (also known as Perlin noise) [20,21] was used to improve the simulation of breast tissue and to represent the breast parenchyma [11,22]. Importantly, the Perlin parameters included a random seed noise generator, ensuring uniqueness for the simulation of each breast parenchyma. More details about the Perlin parameters and database of phantoms are provided in our previous proceeding publications [12,22].

**Figure 1:**
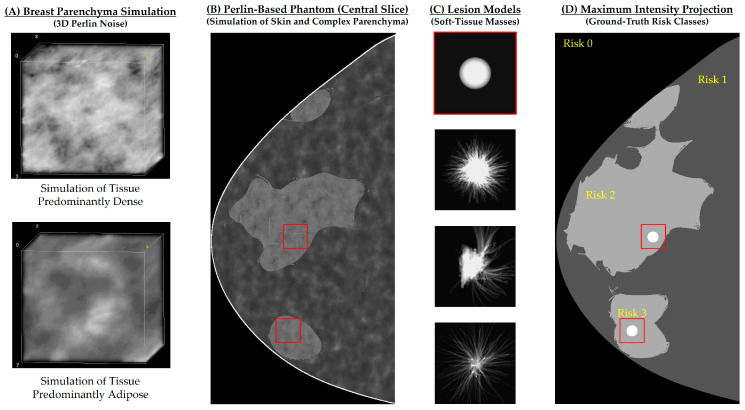
Example of (**A**) complex breast parenchyma and (**B**) Perlin-based phantoms simulated for risk assessment and optimization of DBT acquisitions. (**C**) Lesion models used to simulate risk of (masking) breast cancers. (**D**) Maximum intensity projection (MIP) used as ground truth (3 risk classes) for central DBT projection using the lesion models. Red boxes represent the targeted locations randomly selected for lesion insertion.

Soft-tissue lesions (ellipsoidal and spiculated) were simulated and embedded into the Perlin-noise phantoms (Figure 1). In total, two breast lesions were inserted into each phantom using random positions in the posteroanterior and left–right directions, but always in the center of the phantom in the craniocaudal direction [12]. A voxel additive method [23] was used to insert the lesion models into each phantom (*n* = 264). The attenuation of lesions was controlled by increasing the proportion of dense tissue (*w*) in each voxel [23]. In this study, *w* was set to 0.20 and 0.35 for lesions simulated using the ellipsoidal and spiculated models, respectively. The three lesion models varied in size to closely match those reported by Rafferty et al. ([6, 34] mm) [24]. Lesion models I–IV had dimensions of 7 × 7 × 7 mm^3^, 9 × 8 × 3 mm^3^, 10 × 14 × 4 mm^3^, and 15 × 15 × 4 mm^3^, respectively [25].

### 2.2. Imaging Acquisition and Risk Maps

DBT projections of the breast phantoms were simulated using the OpenVCT framework (University of Pennsylvania, Philadelphia US) [26]. A GPU implementation of the Siddon algorithm [27] was used to project the path and attenuation of each X-ray (i.e., raytracing). The projections were simulated using an acquisition geometry of the NGT system (Table 1). The goal of the VCTs was to demonstrate that areas at high risk of harboring or masking cancers can be detected in a single low-dose projection image, which could ultimately be used to guide the remainder of the scan. The acquisition exposure settings were adjusted to match the thickness and glandularity of each phantom; automatic exposure control data of a clinical system were simulated [28]. The attenuation coefficient data of the materials used to simulate phantoms were taken from the International Commission on Radiation Units & Measurements Report 44 [29].

The maximum intensity projections (MIPs) of the coarse phantoms (i.e., the thresholds of Perlin noise in predominantly adipose and predominantly dense tissues, as shown in Figure 1A) were used to create “risk maps” (Figure 1D). The voxel labels in the phantoms (in order of increasing numerical value) were as follows: air or “background”, “skin”, “adipose”, “dense”, and “lesion”. The MIP operator returned the label with the highest numerical value transected by each ray through the phantom. The MIP image served as the ground truth of the four risk classes used in the multiclass segmentation: background or skin (class 0), predominantly adipose tissue (class 1), predominantly dense tissue (class 2), and lesion (class 3). The MIP images were rescaled to match the dimensions of the DBT central projections.

### 2.3. Multiclass Segmentation

The MIPs were used to train a U-Net [30] model along with the corresponding central DBT projection. The purpose of training the U-Net model in this manner was to demonstrate the feasibility of using a single projection image to identify high-risk areas or areas prone to cancer masking, as this could ultimately be applied to the design of a system capable of real-time image analysis for task-directed scanning motions (self-steering tomosynthesis).

Each central projection image and MIP was cropped to reduce the background and thus the burden of U-Net processing—the cropped region corresponded to the largest phantom AP dimension (20% reduction). Each image and MIP was then downsampled to 360 × 600 pixels.

The U-Net was trained for multiclass segmentation using four risk classes, a batch size of 6, 12 workers (subprocesses used for loading images), a learning rate default of 3 × 10^−4^, and an Adam optimizer. Early stopping with weighted cross-entropy loss (WCEL) was used to optimize the number of epochs (maximum of 250 epochs).

The hyperparameter weights and learning rate were optimized using weights and bias sweeps (WandB v0.12, San Francisco, CA, USA). The model was built using PyTorch 1.10 (LF Projects, LLC, Wilmington, NC, USA) and Python 3.9.9 (PSF, Wilmington, NC, USA). All experiments were conducted on a Dell workstation (Dell Technologies, Round Rock, TX, USA) equipped with two NVIDIA Quadro P5000s (32 GB VRAM), 16 GB DDR RAM, and an Intel Xeon CPU E5-2620 v3 (2.40 GHz, 2401 MHz, six cores).

In total, 168, 24, 24, and 48 input images were used for training, validation, calibration, and testing (~ratio 64:9:9:18%), respectively. For each set, the input images were randomly selected but equally distributed by volumetric breast density, lesion type, Perlin parameters, and breast thickness to avoid bias in the data selection for training, validation, calibration, and testing.

### 2.4. Dirichlet Calibration and Statistical Analyses

The number of instances that were represented as class 3 (i.e., predominantly lesion tissue) was significantly lower than the numbers represented as classes 0 through 2 (background or healthy breast tissue). Each pixel from the input images represented a specific class; the number of instances could not be substantially increased or forcibly simulated to obtain a balanced class-data distribution. Imbalanced class data can result in models for which the overall performance is not representative of the performance for the underrepresented classes. Post hoc Dirichlet calibration (DC) was used to adjust the model’s output probabilities and address potential problems with overconfidence in the predictions [13].

DC provides a calibration map ($\hat{\mu }$) using a vector of class probabilities equal to the softmax (σ) on a linear function of an input probability vector (q), parametrized by a matrix (W) and bias vector (b):

(1) $$\hat{\mu }\left(q;W,b\right)=\sigma (W\ln q+b)$$

The calibration map $\hat{\mu }$ is applied to the vectors of probabilities produced by multiclass models to reduce overconfidence and miscalibrated predictions.

Reliability diagrams [14] were used to visualize and evaluate the current state of calibration of each class predicted by the U-Net model. In these diagrams, the class probabilities are usually binned into *m* equal-width ranges, e.g., for *m* = 10, the bins are [0, 0.1), [0.1, 0.2), …, [0.9, 1.0]. Within $B_{i,j}$, i.e., the *i*-th bin for the *j*-th class, the average probability for class *j*, ${\overset{-}{s}}_{j}$($B_{i,j}$), is compared to the proportion of positives of that class, ${\overset{-}{y}}_{j}$($B_{i,j}$). If the classifier is calibrated for bin $B_{i,j}$, then ${\overset{-}{s}}_{j}$($B_{i,j}$) = ${\overset{-}{y}}_{j}$($B_{i,j}$). In the diagrams, differences between ${\overset{-}{s}}_{j}$($B_{i,j}$) and ${\overset{-}{y}}_{j}$($B_{i,j}$) are represented by error bars. The visual information of a reliability diagram can be aggregated into an overall measure of calibration, called the classwise estimated calibration error (classwise−ECE), given by Equation (2).

(2) $$classwise-ECE=\frac{1}{k}\sum_{j=0}^{k}\sum_{i=1}^{m}\frac{\left|B_{i,j}\right|}{N}\left|{\overset{-}{y}}_{j}(B_{i,j})-{\overset{-}{s}}_{j}(B_{i,j})\right|$$

where *k* and *N* represent the number of classes and instances, respectively, and $\left|B_{i,j}\right|$ represents the bin size.

The performance of the model before and after DC was evaluated using the area under the pooled receiver operating characteristic (ROC) curve (AUC). Two R libraries, “pROC” (version 1.17) and “auctestr” (version 1.0), were used to collect the ROC statistics. The operating point of the ROC curve was defined to be the point that minimized the Euclidean distance relative to the upper left corner of ROC space; at this operating point, we calculated the true positive rate (TPR), true negative rate (TNR), positive predictive value (PPV), and negative predictive value (NPV). Two segmentation metrics, Jaccard (Jac) and Dice coefficients, were also calculated using the four class predictions.

### 2.5. Identification of Suspicious Findings

We also evaluated the accuracy of the U-Net model in identifying suspicious findings. We defined a suspicious finding as a cluster of pixels labeled as lesion (i.e., class 3) in the MIP. A suspicious finding was correctly identified (true positive or TP) when the region predicted as being class 3 overlapped a cluster of class 3 pixels in the ground truth. Analogously, false positive (FP) findings had no overlap between the predicted class 3 region and any class 3 cluster in the ground truth. False negative findings occurred when none of the pixels in a class 3 cluster in the ground truth were predicted as being in class 3. True negative (TN) findings were not evaluated as this was a lesion identification task; this analysis differed from the preceding evaluation of class segmentation (Section 2.5). The TP, FP, and FN findings were calculated for each test image before and after DC.

Softmax was used to obtain the vectors of probabilities of each test image before and after DC. Free-response ROC (FROC) analyses [31] were performed by thresholding the probabilities (from 1.00 to 0.00 in 0.01 steps) and by calculating the sensitivity of findings localized and the number of FP findings per thresholded image. The TP and FP findings were identified using a postprocessing technique based on connected components in the thresholded images [32]. The ground-truth images (class 3) were used to classify the identified findings in TP and FP. FROC curves were created using the fraction of TP findings as a function of the average number of FP findings per image.

## 3. Results

### 3.1. U-Net Segmentation and Dirichlet Calibration (DC)

**Figure 2:**
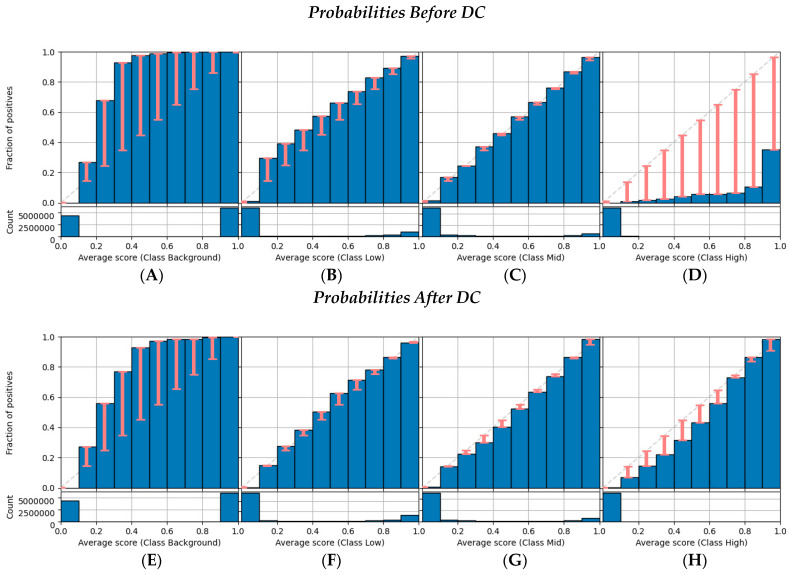
Reliability diagrams before and after DC for classes (**A**,**E**) *j* = 0, (**B**,**F**) *j* = 1, (**C**,**G**) *j* = 2, and (**D**,**H**) *j* = 3.

The reliability diagrams of each class are shown in Figure 2. Before DC, the U-Net model demonstrated overconfidence, resulting in a disproportionate likelihood of predicting the highest class (*j* = 3). Before DC, in the last bin for class *j* = 3 (Figure 2C), the average probability, ${\overset{-}{s}}_{3}$(*B*_10,3_) ≈ 0.95, was significantly higher than the observed proportion of positives (${\overset{-}{y}}_{j}$(*B*_10,3_) ≈ 0.4). The reliability diagrams show that calibration error was reduced significantly after DC, especially for class *j* = 1 (Figure 2F) and *j* = 3 (Figure 2G).

### 3.2. ROC Analyses

**Figure 3:**
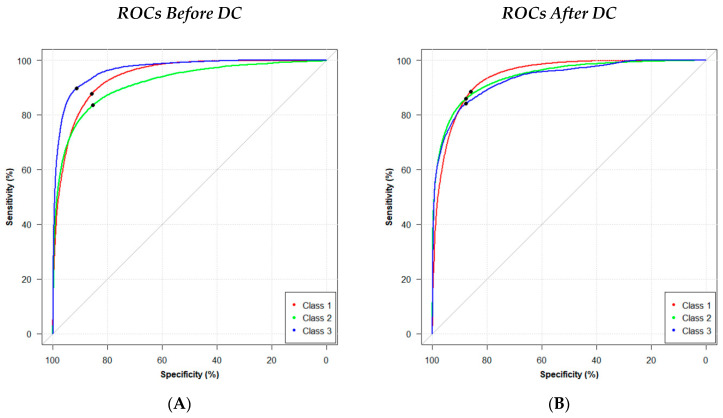
ROC curves (**A**) before and (**B**) after DC. Optimal operating points are indicated in black.

ROC statistics were collected (Figure 3) and a summary of the segmentation and classification metrics is shown in Table 2. Before DC, the model resulted in AUC values of 0.94, 0.92, and 0.90 for classes 1, 2, and 3, respectively. After DC, the model obtained an improvement in the segmentation of classes 2 (AUC = 0.94) and 3 (AUC = 0.93); no change in performance was observed for the segmentation of class 1. We also observed a decrease in both TPR (0.90 vs. 0.84) and TNR (0.91 vs. 0.88) after DC for class 3. However, it was noted that DC substantially improved the Dice (0.28 vs. 0.43) and Jaccard (0.16 vs. 0.28) segmentation metrics.

### 3.3. Lesion Identification and FROC Statistics

**Figure 4:**
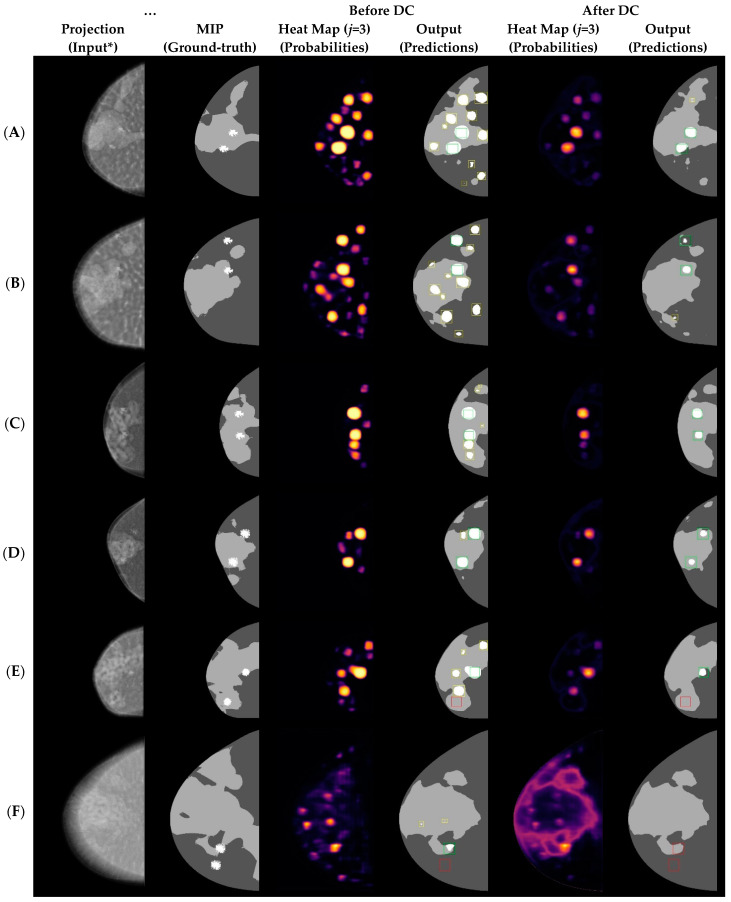
Results obtained from U-Net segmentation before and after DC. CBT and CND in the examples are (45.0, 93.0), (55.0, 109.0), (35.0, 59.0), (35.0, 64.1), (45.0, 77.7), and (75.0, 125.6) mm, for (**A**–**F**), respectively. Green, yellow, and red pixels represent TP, FP, and FN predictions, respectively. * Projections processed with minimum filtering only for data visualization (Adara software, Real-Time Tomography, Villanova, PA, USA).

DC improved the performance of the multiclass segmentation explored in this work (Figure 4). Before DC, we observed that the U-Net model had a high rate of FP predictions for class 3. DC resulted in a significant reduction in the number of FPs with a small increase in FN predictions of suspicious regions (Figure 4F and Table 3). Most FN predictions occurred in thicker or larger breast phantoms. In total, after DC, 11 out of 19 FN predictions occurred in breasts with CBT > 65 mm (mean CND = 104.3 mm). The images in which both lesions were missed were those in which CBT > 75 mm or CND > 110 mm (*n* = 4). Importantly, by examining the maximum probability of the vectors obtained by the softmax (i.e., the peak probability value of class 3 after DC), additional lesions could be identified, potentially reducing the number of FN findings. For example, in Figure 4F, one lesion was associated with the maximum probability in the image, but it fell below the threshold for identification.

**Figure 5:**
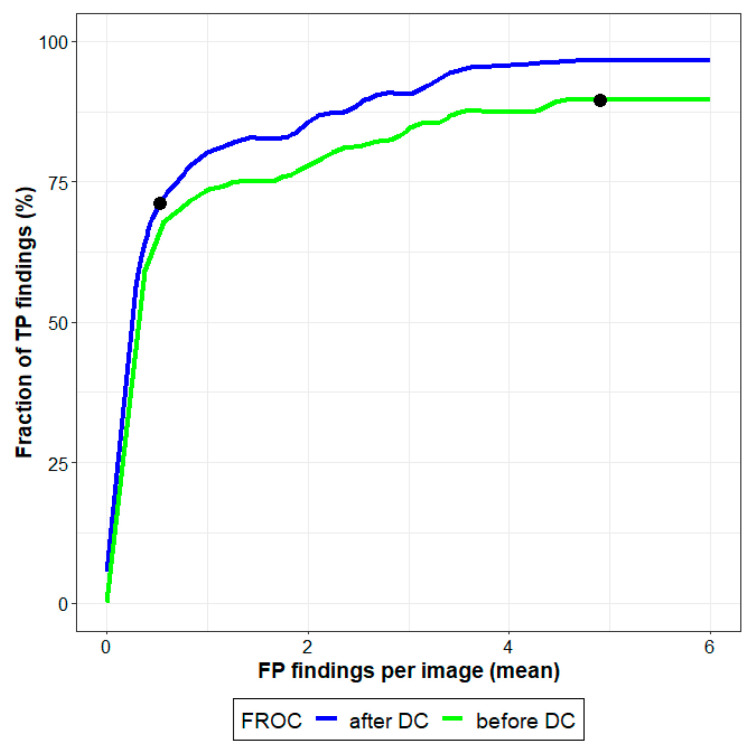
FROC curves before and after DC. FROC curves were used for the identification of suspicious regions using vectors of probabilities. TP findings (%) as a function of mean FP findings of predictions are highlighted in black.

FROC curves were used to evaluate the performance of the U-Net model before and after DC. The FROC curves demonstrated an improvement in performance after DC with a substantial increase in sensitivity at a given FP rate (Figure 5). After DC, at 2 FPs/image, the sensitivity was 81.3%; before DC, the sensitivity was only 76.0%. At 5 FPs/image, the sensitivity was 96.6% with DC, and 89.9% otherwise.

## 4. Discussion

DBT systems acquire images over a limited angular range with a limited number of projections; the resulting datasets are undersampled and produce out-of-plane artifacts in the reconstructions that may compromise the detectability of lesions. Our previous work with virtual phantoms demonstrated that there is benefit to customizing the scanning motion based on breast size, but it did not consider the impact of lesion detectability [6]. Our long-term goal is to customize the scanning motion based on the location of suspicious regions, such as clinical findings or areas susceptible to cancer masking, in an effort to improve both the sensitivity and specificity of DBT. The method of detecting these suspicious regions should be robust to breast size and shape, as well as the complexity of the internal breast composition.

Using a VCT-based method, this study demonstrated the feasibility of using the central (low-dose) projection image to segment the breast into various classes of tissue for a variety of breast sizes and compositions. Regions of low or high risk can be segmented accurately from simulated low-dose projections of complex breast parenchyma. ROC statistics were collected to evaluate changes in performance before and after DC. We achieved AUC values of 0.93, 0.94, and 0.94 for the segmentation of classes 1, 2, and 3, respectively. To obtain these results, optimization and calibration methods were required to address problems of overconfidence and training with imbalanced class data.

There was a clear trade-off between the reduction of FPs and an increase in FN findings. We showed using computer simulations that DC is a good method to optimize the identification of high-risk areas cost-effectively using low-dose projections, substantially reducing the number of FP findings. This was expected because, in our task, DC had the effect of decreasing the confidence of some high-risk areas. Nevertheless, given the calibrated probabilities, a possible next step would be to find optimal decision thresholds based on the costs of misclassification for each class [33]. The thresholds could also be varied as a function of breast size and volume to accommodate population-based differences in the risk of masking. Finally, when applying DC, we considered that every pixel was independent, which does not happen in practice, given that nearby pixels tend to be assigned to similar classes. Thus, in the future we will investigate calibration methods that consider pixel neighborhoods and different image regions.

FROC analysis helped us to better understand the sensitivity of localizing lesions (TP findings) and the costs associated with FP findings. After DC, the FROC curve showed a higher sensitivity and specificity when compared with the curve before DC. In the future, we will perform more detailed statistical analyses of TP rates and the costs associated with FP findings.

Although this study focused solely on the NGT system, in future work we would like to apply the multiclass U-Net model to clinical DBT systems. We will fine-tune our proposed models using clinical data collected retrospectively, and, ultimately, the domain of virtual models will be adapted to clinical data (effectively making the transition from the virtual to the real world). We will also explore additional calibration methods and customizations in the U-Net architecture to improve the performance of this model.

Alternate loss functions and additional AI architectures could be investigated to evaluate further the performance of the proposed segmentation method. Abraham and Khan proposed a loss function to address imbalanced class data for imaging segmentation [34,35]. We have preliminary data showing that the focal Tversky loss (FTL) function may not result in the outright highest precision or recall rates [36]; however, because of its nonlinear nature, FTL provides better control and balance between FP and FN predictions. In our future work, we could compare models developed with WCEL + DC and FTL + DC.

This study had some limitations. The simulation of mammary parenchyma with Perlin noise does not fully simulate the nuances and fine structures found within the breast. The noise parameters still need to be fine-tuned in future work to improve the representation of breast parenchyma [11,37,38]. In a previous review article, Marshall and Bosmans emphasized that the degree of realism required for breast imaging is somewhat open and subjective [39]. We acknowledge that the Perlin noise parameters could be further optimized by validating the realism of the imaging data using human readers. However, there are other methods besides subjective visual inspection to support the assessment of the representation of simulated breast parenchyma as compared with real patient images [39]. In our previous work, power spectrum [40] and Laplacian fractional entropy [41] were used to evaluate the realism and quality of imaging data simulated with Perlin noise [11]. These metrics allowed us to quantify realism objectively and compare the anatomical noise structures found in the mammary parenchyma; the same noise parameters were used to simulate images in this work.

Only four lesion models were used and embedded into the simulated parenchyma; additional lesions that vary in size and composition must be simulated to support the results obtained in this work. However, it is important to note that the clinical task represented in this work does not involve the characterization of breast lesions or classification of abnormalities. Instead, the clinical task is fundamentally different; the proposed AI model will ultimately identify *suspicious areas that require increased scrutiny by the observer*; these include possible areas of cancer, architectural distortions, other suspicious findings, or clustered dense tissue which could mask cancers. This work is a proof of concept to evaluate the feasibility of using a single low-dose projection to customize and guide the NGT system for subsequent projections.

We also acknowledge that the ground-truth risk estimates used to train the U-Net model can be improved. The MIP will preserve the location and shape of lesions (e.g., small spiculations), but it can overestimate the distribution of dense tissues (defined as class 2). Furthermore, precisely segmenting images to define either dense regions or regions with a high risk of masking breast cancers is somewhat subjective [42]; however, this imprecision is present in both phantom and clinical images. This subjectivity supports the need for calibration methods, as we have shown that DC can improve predictions, leading to more cost-effective segmentation.

Finally, it should be emphasized that the segmentation method described in this paper is not intended for use in a computer-aided detection (CAD) system per se, but instead in a self-steering tomosynthesis system where the goal is to direct the scanning motion around an area under suspicion. Our results support the feasibility of detecting suspicious areas in a single projection image, beginning at the start of the scan. Ultimately, the determination of suspicious regions could be used to direct the remainder of the scanning motion around these areas. Although the NGT system is not yet capable of customized scanning motions, our long-term goal is to utilize these customized motions clinically. Note that the development of task-directed scanning motions is beyond the scope of this paper. Our future work will investigate whether customizing the scanning motion around suspicious area(s) in the breast offers improvements in image quality and lesion detectability.

## 5. Conclusions

Calibration can address problems with overconfidence of segmentation models and imbalanced class data used for training U-Nets. The segmentation of risk-classified areas from computer-simulated low-dose projections improved after DC, resulting in a substantial reduction of FP predictions of suspicious findings.

Using VCTs, we demonstrated the feasibility of detecting suspicious areas in the breast in a low-dose projection image. The ultimate goal of this work is to apply this detection method to develop and use customized scanning motions in an NGT system capable of self-steering tomosynthesis.

## Figures and Tables

**Table 1:** Parameters used to simulate acquisition geometry of the NGT system.

Radiation exposure (mode)	AEC
Detector size (width × height, mm)	239.36 × 304.64
Detector type (detector motion)	a-Se (stationary)
Detector element size (width × height, mm)	0.085 × 0.085
Source image distance (mm)	738.01
Target/filter combination (X-ray tube motion)	W/Al (step-and-shoot)
Reconstructed voxel size (width × height, mm)	0.085 × 0.085
Imaging processing	None (raw)

**Table 2:** Summary of segmentation (Dice and Jac) and classification (AUC, TPR, TNR, PPV, and PNV) metrics calculated (A) before and (B) after DC.

(A)								(B)						
Class	Dice	Jac	AUC	TPR	TNR	PPV	NPV	Dice	Jac	AUC	TPR	TNR	PPV	NPV
0	1.00	0.99	-	-	-	-	-	1.00	0.99	-	-	-	-	-
1	0.89	0.79	0.94	0.88	0.86	0.90	0.84	0.90	0.81	0.94	0.89	0.86	0.90	0.85
2	0.82	0.69	0.92	0.84	0.85	0.80	0.88	0.85	0.73	0.94	0.86	0.88	0.83	0.90
3	0.28	0.16	0.90	0.90	0.91	0.10	0.99	0.43	0.28	0.93	0.84	0.88	0.07	0.99

**Table 3:** Identification of suspicious regions (A) before and (B) after DC using output predictions. TNs were not applicable (NA) in this analysis.

…	(A)		(B)	
	Positive	Negative	Positive	Negative
Predicted Positive	85	236	69	24
Predicted Negative	11	NA	27	NA

## Data Availability

The data generated during the current study are available from the corresponding author on reasonable request.
